# Occult HBV Infection in Patients Infected by HIV or HCV: Comparison between HBV-DNA and Two Assays for HBsAg

**DOI:** 10.3390/v16030412

**Published:** 2024-03-07

**Authors:** Silvia Meschi, Klizia Mizzoni, Bruno Daniele Leoni, Claudio Galli, Anna Rosa Garbuglia, Stefano Belladonna, Enrico Girardi, Fabrizio Maggi, the HBSAGN Study Group

**Affiliations:** 1 Laboratory of Virology, National Institute for Infectious Diseases Lazzaro Spallanzani IRCCS, 00149 Rome, Italyannarosa.garbuglia@inmi.it (A.R.G.); fabrizio.maggi@inmi.it (F.M.); 2 Validation & Verification, Core Laboratory, Abbott, 00144 Rome, Italy; 3 Independent Researcher, 00139 Rome, Italy; claudiogalli26@gmail.com; 4 Abbott Molecular, Abbott, 00144 Rome, Italy; 5 Scientific Direction, National Institute for Infectious Diseases Lazzaro Spallanzani IRCCS, 00149 Rome, Italy

**Keywords:** occult hepatitis B (OBI), HBV-DNA, anti-HBc, HBsAg, HCV, HIV

## Abstract

We investigated the frequency and serological correlates of occult hepatitis B virus infection (OBI) and the potential impact of a highly sensitive assay for HBsAg in subjects infected by human immunodeficiency virus (HIV) or hepatitis C virus (HCV), who are also at risk for hepatitis B virus (HBV) infection, often in an occult form. Samples from 499 patients with HIV, all HBsAg negative and anti-HBc positive, and 137 patients with HCV were tested for HBV-DNA, anti-HBc, anti-HBs, and HBsAg by a conventional and highly sensitive assay. HBV biomarkers were detected in 71.5% of HCV-RNA-positive, with a higher prevalence of cases positive only for anti-HBc in patients with HCV than in those with HIV. HBV-DNA was detectable in 0.6% of HIV-positive and 7.3% of HCV-RNA-positive patients. Among patients with HCV, four were positive for HBsAg and negative for HBV-DNA, bringing the rate of HBV-active infection in this group to 10.2%. Active HBV infection was not related to gender or specific patterns of HBV biomarkers but was higher in HCV patients coinfected by HIV compared to those infected only by HCV. Monitoring patients at high risk for HBV infection and reactivation may require testing for both HBV-DNA and HBsAg.

## 1. Introduction

Occult hepatitis B virus (HBV) infection (OBI) has been defined as the detection of replicative HBV-DNA in the liver and/or in the blood without detectable HBV surface antigen (HBsAg) [1]. This phenomenon was initially considered a casual observation of uncertain clinical significance [2], though its frequency could be high in patients with concurrent infections, such as hepatitis C virus (HCV) and/or human immunodeficiency virus (HIV) infection [3]. Further studies on the replicative mechanism of HBV have helped to elucidate the genesis and significance of OBI. After HBV enters the hepatocyte, viral replication includes the production of an episomal form of HBV-DNA (covalently closed circular DNA, or cccDNA). This episomal HBV DNA, which is in fact a viral minichromosome, is stable and persists in the nucleus of infected cells after the apparent recovery from acute hepatitis B, diagnosed by the negativization of HBsAg and seroconversion to anti-HBs. The persistence of cccDNA along with the long life of hepatocytes implies that HBV infection usually persists for a lifetime, despite efficient control by the host immune system. The majority of OBI cases have low levels of HBV cccDNA in the liver, and the host’s immunological and epigenetic mechanisms suppress replication activity and viral protein expression. Since in OBI, the levels of transcriptionally active cccDNA are low, HBV-RNA transcription and the ensuing protein translation and expression are limited, and this results in a lack of detectable circulating HBsAg. In addition to cccDNA, the persistence of a viral infection after spontaneous or treatment-related clearance of HBsAg is also sustained by the partial integration of HBV-DNA in the cell genome.

OBI shall be considered a temporary balance between the virus replication capability and the host immune response [1]. In immunocompetent individuals, the infection persists without clinical signs or symptoms, with a low but not negligible rate of progression to chronic liver disease [1,4]. On the other hand, patients with a concurrent HCV infection may show a reactivation of OBI during or after treatment with directly acting antivirals (DAAs) [5] and patients with immunosuppression, either caused by human immunodeficiency virus (HIV) infection or subjected to immunosuppressive treatment after solid organ or bone marrow transplantation, have a higher likelihood of experiencing a reactivation of the latent HBV infection that may cause a severe and sometimes fatal liver disease [1]. In this study, we aimed to investigate the frequency and serological correlates of OBI in two cohorts of subjects infected by HIV and HCV, respectively, and to evaluate the sensitivity of a new device for the detection of HBsAg in those patients.

## 2. Materials and Methods

### 2.1. Ethical

The study was carried out on fully anonymized, repository surplus serum or plasma specimens stored at −20 °C or lower at the Laboratory of Virology of the National Institute for Infectious Diseases “Lazzaro Spallanzani” IRCCS (INMI) in Rome. The INMI Ethics Committee authorized the study (approval number: no. 28-March 1st, 2019), which waived patient notification if samples had been anonymized and could not be traced back to individual patients.

### 2.2. HBV-DNA Test

All serum samples with sufficient volume were assayed retrospectively for HBV-DNA by a quantitative method (Real-Time HBV-DNA, Abbott Molecular, Des Plaines, IL, USA) with a limit of quantitation of 30 international units (IU)/mL.

### 2.3. Serological Tests

The study samples were selected from three different patient groups.

Group A. We aimed to enroll 500–600 patients, aged 18 years or more, who were routinely followed up at our institution for a positive HIV status. Patients were eligible if they were negative for HBsAg by the assay currently in use (ARCHITECT HBsAg II, Abbott Diagnostics GmbH, Wiesbaden, Germany) and positive for hepatitis B core antigen (anti-HBc), with or without protective antibody to HBsAg (anti-HBs). A single serum or plasma sample from each patient was assayed for HBsAg by a newly developed test with enhanced sensitivity (ARCHITECT HBsAg NEXT qualitative, Abbott GmbH, Wiesbaden, Germany). The main difference between the two assays relates to the analytical sensitivity, which corresponds to 0.05 international units (IU)/mL for HBsAg II and to 0.005 IU/mL for HBsAg NEXT [6,7]. Briefly, HBsAg NEXT is a one-step chemiluminescent microparticle immunoassay with two monoclonal antibodies coated on the microparticles and a goat anti-HBs conjugate. The results were expressed as a sample-to-cutoff ratio (S/CO). The positivity for HBsAg in HBsAg NEXT reactive samples (S/CO > 1.00) was confirmed by a neutralization assay (ARCHITECT HBsAg NEXT qualitative confirmatory assay, Abbott GmbH, Wiesbaden, Germany). Samples were also tested for anti-HBc and anti-HBs by the respective Abbott ARCHITECT assays if the historical results were obtained >6 months before enrollment.

Group B. We aimed to enroll 120–150 patients, aged 18 years or more, who were followed up at our institution for an active HCV infection established according to a positive result for HCV-RNA. A single specimen from each patient was tested for HBsAg by both HBsAg II and HBsAg NEXT, for anti-HBc and anti-HBs as described above.

Group C. To further check the sensitivity of the HBsAg NEXT assay compared to the standard version of the assay and to HBV-DNA positivity, we also aimed to include a few routine repository samples with low levels (<30 to 1500 IU/mL) of HBV-DNA and a negative result for HBsAg by the assay currently in use, which are most often detected in OBI [1,7].

### 2.4. Statistical Analysis

The data analysis, performed separately on groups A and B, included demographics (gender, age), patterns of HBV serological markers, frequency of OBI (i.e., positivity for HBV-DNA), and positivity rates by the HBsAg NEXT assay. The concordance between HBsAg tested by the newly developed test and HBV-DNA, the relationship between OBI and/or HBsAg NEXT positivity, and the different serological patterns of HBV infection (positivity for anti-HBc and anti-HBs, alone or combined) were also evaluated. Finally, we carried out a separate analysis on subjects from group B for whom the HIV status was known, evaluating separately the HBV biomarker status on the HIV-negative or HIV-positive groups with or without a suppression of HIV replication, defined by HIV-RNA levels <50 copies/mL [8].

The statistical analysis and descriptive results are presented as mean ± standard deviation (SD) for continuous variables, medians with Q1 and Q3 when variables are non-normally distributed, and as a percentage (%) for categorical variables. Differences in categorical variables between groups were evaluated with Pearson’s chi-squared or Fisher’s exact test according to the sample size. Multiple comparisons in a non-normal distribution were performed with the Steel–Dwass–Critchlow–Fligner pairwise ranking nonparametric method.

A *p*-value < 0.05 was considered statistically significant, and the sample size was chosen for having a statistical power ≥0.8. The dataset was built in Microsoft Excel (Microsoft Corporation, Redmond, WA, USA) and the statistical analysis was performed with Analyze-it for Microsoft Excel 4.92.4 and R software version 3.6.0 “https://www.R-project.org (accessed on 22 May 2019)”.

## 3. Results

### 3.1. Group A—HIV-Positive Patients

**Figure 1:**
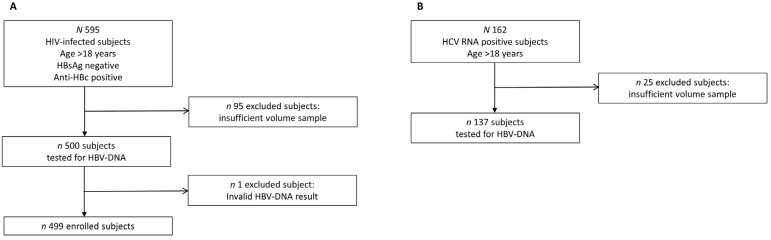
Flowchart for study selection. (**A**): group A, subjects with HIV; (**B**): group B, subjects with HCV. Boxes and arrows represent the subgroup populations.

The initial selection included 595 patients. We could analyze HBV-DNA only on 500 samples due to insufficient volume on the other 95 and obtained an invalid result on one, so the final enrollment included 499 patients, 83.9% of the initial cohort (Figure 1A). The study population included 81 females (16.2%) and 418 males (83.8%). The demographic characteristics of patients belonging to group A are shown in Table 1.

This distribution reflects the gender distribution of HIV-positive patients attending our institution. The median age (53 years) and age distribution did not differ significantly between genders, nor did the percentage of HIV-suppressed cases (414, or 83%; 74.1% among females and 84.7% among males) (Table 2).

#### 3.1.1. HBV Biomarkers

Fifteen subjects (12 males, 3 females; 3%) were negative for anti-HBc upon testing on the study sample, despite having been recorded as positive on prior testing. We kept those samples anyway since the enrollment criteria based on historical data were met and included those in the data analysis for HBV biomarkers, HBsAg Next, and HBV-DNA. Out of those 15 subjects, 11 cases (2.2% of the total) were positive for anti-HBs as defined by levels >10 mIU/mL [9]. Of the remaining samples, 379 (76.0%) were positive for both anti-HBc and anti-HBs, and 105 (21%) were positive only for anti-HBc. The anti-HBc positivity without the presence of anti-HBs in negative subjects was significantly different between genders (25 females, 30.8% vs. 80 males, 19.1%; *p* = 0.03) (Table 2).

#### 3.1.2. Positivity by HBV-DNA and HBsAg Next Assays

**Figure 2:**
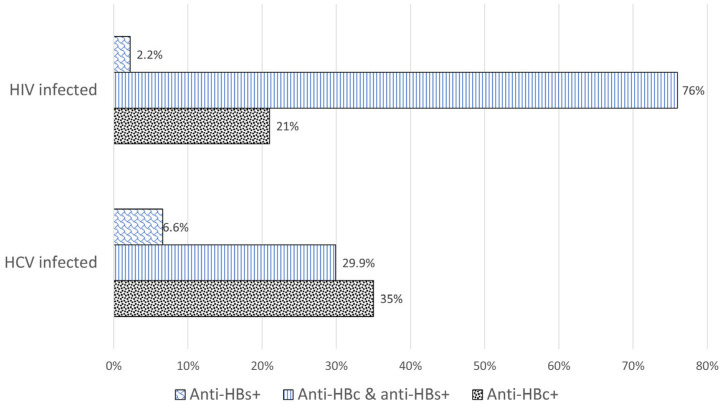
Frequency of serological patterns of HBV infection in 499 patients with HIV and 137 patients with HCV. Four patients with HIV and thirty-nine patients with HCV negative for all HBV markers were not included. The difference in anti-HBc-only positive samples between the two groups was highly significant (*p* < 0.005).

Only three specimens (0.7%) were positive for HBV-DNA, two with levels <30 IU/mL (DNA detected but not quantified) and one with 67 IU/mL (Table 3 and Figure 2).

None of the three was positive for HBsAg by the HBsAg NEXT assay, which gave no reactive result on any sample from this group.

### 3.2. Group B—Patients with HCV

The initial selection included 162 patients. We were unable to test for HBV-DNA on 25 samples, and the final enrolment was 137 patients, 84.6% of the initial cohort (Figure 1B), of whom 50 were females (36.4%) and 87 were males (63.6%). The median age (54 years) and age distribution did not differ significantly between genders, nor did the levels of HCV-RNA (median: 5.75 log10 UI/mL; 5.83 log10 UI/mL in females, 5.71 log10 UI/mL in males) (Table 2).

#### 3.2.1. HBV Biomarkers

No specimen was positive for HBsAg by the HBsAg II assay. Thirty-nine samples (28.5%) were negative for all HBV biomarkers, with a significantly higher rate among females (44% vs. 19.5%; *p* < 0.1). Of the remaining samples, 48 (35.0%) were positive only for anti-HBc, 9 (6.6%) were positive only for anti-HBs, probably due to vaccination, and 41 (29.9%) were positive for anti-HBc and anti-HBs (Table 1).

#### 3.2.2. Positivity by HBV-DNA and HBsAg Next Assays

Ten specimens were positive for HBV-DNA (7.3%), with levels <30 IU/mL (detected but not quantifiable) in six patients and ranging between 48 and 133 IU/mL (Table 3) in the remaining four patients. None of those was positive by either HBsAg assay.

Seven samples were initially reactive to the HBsAg Next assay, but only four samples were confirmed (2.9%). Two samples were positive for anti-HBc, and two samples were reactive for anti-HBs and anti-HBc (Table 3).

#### 3.2.3. Patients with HCV/HIV Coinfection

Historical data regarding HIV infection were available for eighty-eight patients (64.2%) from group B; 72.4% were males and 50% were females (*p* < 0.01). The overall positivity rate for HIV infection was 61.4% and higher in males than in females (65.1% vs. 52%), although it was not statistically significant. Viral replication was suppressed in the majority of HIV positives (39, 72.2%). HBsAg NEXT was positive in two samples from HIV-suppressed patients (one male and one female), in one sample from a not-suppressed male patient, and in one HIV-negative male patient.

### 3.3. Group C—Patients with Low HBV-DNA

Eight samples from patients with low HBV-DNA levels were retrieved. HBV-DNA levels ranged from <30 to 1186 IU/mL. Three patients were HIV-negative and five were positive (one suppressed, four not suppressed). Three subjects were positive for anti-HBc and anti-HBs, and five were positive for anti-HBc only. None was positive by the HBsAg II assay, and two were confirmed positive by the HBsAg NEXT assay; one subject was positive for anti-HBc and anti-HBs, and one was positive for anti-HBc only (Table 3). Demographic data and testing results for patients belonging to group C are described in Table S1.

By contrast, the patterns of HBV serological biomarkers between the whole set of patients from group A and patients from group B who tested positive for at least one biomarker were different (Figure 2). The frequency difference of samples positive for anti-HBc and anti-HBs (76% among HIV-infected vs. 29.9% among HCV-infected) was highly significant (*p* < 0.001), and, conversely, the relative frequency of samples positive for anti-HBc was only significantly higher in patients with HCV than in patients with HIV (35% vs. 21%; *p* < 0.005). There was no clear gender-related difference, as anti-HBc positives were only higher in HIV-positive females but lower in HCV-positive females (Table 2).

**Figure 3:**
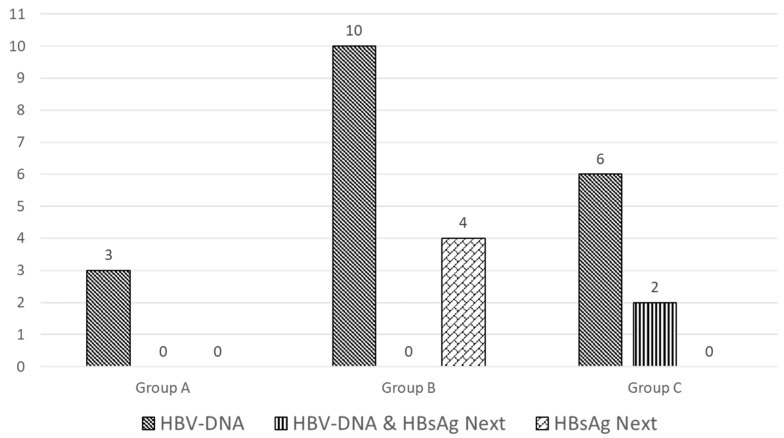
Positivity for HBV-DNA and/or for HBsAg by the HBsAg NEXT assay in three groups of subjects. Group A = subjects with HIV; group B = subjects with HCV; Group C = subjects with low HBV-DNA levels. The frequency of positivity for HBV-DNA in groups A and B was 0.6% and 7.3%, respectively (*p* < 0.0001).

Overall, 21 out of 644 samples were positive for HBV-DNA and 6 for HBsAg by the NEXT assay, but the concordance between those two biomarkers of HBV replication was low since only two specimens were positive for both biomarkers (Table 3 and Figure 3).

No significant difference in the frequency of active HBV infection, i.e., positivity for HBV-DNA and/or HBsAg, was observed between genders or according to the patterns of HBV biomarkers, nor the status of HIV infection.

## 4. Discussion

Nowadays, OBI is considered a natural evolution of HBV infection in patients who have recovered, having cleared HBsAg and developed a protective immune response through the clonal expansion and maturation of B lymphocytes, which leads to the detectable presence of anti-HBs [10]. OBI is defined by the presence of replication-competent HBV-DNA in the liver and/or in the blood, but this biomarker is detectable in serum or plasma to different degrees and at different rates depending on several factors, such as the population examined, the analytical sensitivity of the assay employed to detect HBV-DNA, and timing, i.e., if samples obtained at different time points are tested. The last issue is especially relevant since many studies have demonstrated that HBV-DNA may be detected only intermittently in serum or plasma, and the levels are usually very low—about 1000 copies/mL or less [11,12]. OBI being potentially a lifetime phenomenon, testing for anti-HBc, which is the more stable marker of an HBV infection, may be considered a surrogate strategy to detect it, but this is both redundant and insufficient. Redundancy stems from the fact that in most subjects who test positive for anti-HBc, HBV-DNA cannot be detected. On the other side, testing for anti-HBc may fail to detect subjects with an OBI, since 1% to 20% of OBI cases are seronegative for all HBV biomarkers, either because they have lost anti-HBs and anti-HBc antibodies over time (or, better, the levels of those antibodies have fallen below the detection limit of currently available assays) or may never have developed those antibodies. In people with seropositive-OBI, HBsAg may have become negative either following the resolution of acute hepatitis B (thus, after a few months of carrying HBsAg) or after decades of HBsAg-positive (namely, “overt”) chronic HBV infection with or without disease. It is unknown whether patients with chronic HBV infection or disease who become HBsAg negative following antiviral therapy are comparable to patients who spontaneously clear HBsAg from the virological and immunological points of view, e.g., the duration of exposure to a high viral load and restoration of the immune response to HBV. The possible clinical implications of this distinction are also unknown.

The diagnosis of OBI is challenging because it relies on the detection of HBV-DNA either in the liver, which requires a liver biopsy and cannot be achieved by standardized assays [11], or in plasma or serum. In OBI, HBV-DNA usually circulates at very low levels in plasma or serum, often <100 UI/mL [1,12], and shows fluctuations that may lead to a negative result even if highly sensitive HBV-DNA assays are employed [12,13]. Testing for anti-HBcs is also recommended, as those are usually detectable in all phases of the infection after the first few weeks [9]. Anti-HBc positivity was identified as an independent risk factor for the development of hepatocellular cancer (HCC) [9], and most subjects with an OBI are positive for anti-HBc. On the other side, the frequency of HBV-DNA positivity in patients positive for anti-HBc and negative for HBsAg is low [12,13], and in some instances, a virological reactivation of HBV has also been documented in anti-HBc-positive individuals with undetectable HBsAg and HBV-DNA [1]. In recent years, efforts have been made to increase the sensitivity of HBsAg assays to allow a better detection of active HBV infection, and assays of the latest generation can detect 0.005 IU/mL of HBsAg, which has been estimated to correspond to 30–40 IU/mL of HBV-DNA [6,7]. In this study, we challenged one of those new assays on two different scenarios in which an OBI has a definite clinical relevance: patients with HIV positive for anti-HBc and patients with HCV. Among patients with HIV, OBI frequency was very low (three subjects, 0.6%), and in all three, the HBV load was lower than 100 IU/mL, so it is not surprising that HBsAg was negative by both the conventional and the highly sensitive assay. The very low ratio of active HBV is most likely linked to the fact that most of those patients were HIV-RNA-suppressed due to antiretroviral treatment, as most current drug regimens for HIV treatment, such as lamivudine, tenofovir disoproxil fumarate, and tenofovir alafenamide, are also active against HBV [1,14,15,16]. The occurrence of OBI reactivation in people infected by HIV is uncommon, usually below 0.02 cases per 100 persons-year [17,18]. Nevertheless, due to the severity of the potential consequences of HBV infection in HIV-positive patients [17], HBV biomarkers shall always be monitored in patients with severe immunodeficiency and/or on ART regimens that do not include tenofovir or lamivudine [1,18,19].

Our cohort of patients with HCV showed a high rate of HBV infection, as previously described [4,5,20]. Those patients showed a higher rate of OBI compared to HIV-positive individuals (Table 1) and a much higher frequency of HBV active infection, either OBI or overt (HBsAg-positive), compared to subjects with HIV (10.2% vs. 0.6%; *p* < 0.001). From a virological and clinical standpoint, in most cases, HCV/HBV coinfection appears to inhibit HBV replication, and accordingly, clearance of the HCV infection, either spontaneous or driven by treatment with IFN-based regimens or with DAAs, can be associated with HBV reactivations leading to hepatitis flares [21].

The spectrum and features of HBV Infection in patients with an active infection by HCV require attention since current data and evidence have shown a non-negligible incidence of HBV reactivation in patients with chronic hepatitis C when treatment with directly acting antivirals (DAAs) is started to eradicate HCV and during the follow-up period after treatment [9,21,22]. The spectrum of clinical manifestations associated with HBV reactivation is wide, ranging from laboratory abnormalities with minimal evidence of liver disease to life-threatening hepatitis that is more frequent in HBV-endemic areas. The rate of reactivation depends on the DAA regimen, the frequency and duration of follow-up testing after treatment, and the presence or absence of HBsAg at baseline, thus varying according to the sensitivity of the diagnostic method employed to test for this biomarker. Based on these assumptions and according to the current recommendations, all patients with diagnosed HCV infections planned for DAA treatment should be screened for HBsAg. Coinfected individuals with evidence of a chronic liver disease sustained by the HBV infection, thus fulfilling the standard criteria for HBV treatment, should be treated with nucleoside/nucleotide analogs (NUCs), while HBsAg-positive patients not meeting the criteria for anti-HBV treatment should receive prophylaxis with the same drugs before starting DAA treatment for hepatitis C and should be monitored for HBV reactivation for at least 12 weeks after completion of anti-HCV therapy. The need to monitor those patients with methods with a high sensitivity for an active HBV infection is quite evident, and the currently available HBV-DNA assays fulfill that need. However, quite interestingly, four subjects were confirmed positive for HBsAg with an undetectable HBV-DNA. This may depend on the different kinetics of and fluctuations in the two parameters [21], but a limitation of our study is that its cross-sectional nature did not allow us to ascertain the behavior of HBsAg and HBV-DNA over time. Our results suggest that monitoring HCV in patients coinfected by HBV using both HBV-DNA and HBsAg via highly sensitive assays shall be strongly recommended.

Of note, at least 54 of the HCV-positive patients included in this survey were also infected by HIV, most likely sharing common risk factors; unfortunately, we could not investigate those nor establish a temporal sequence for the acquisition of those infections. In group B, HBV-DNA was detected only among HCV-HIV coinfected (4.6%), and three out of four subjects positive for HBsAg by the NEXT assay were infected by HIV. Though this may suggest that HIV-driven immunosuppression may enhance HBV replication, we acknowledge a limitation there, since not all patients with HIV had been tested for HIV.

In general, though those data allow some interesting speculations, the major limitation of our study is that our results need to be confirmed on a greater number of cases, both from individuals with HIV and HCV, to reach more definite conclusions. Another limitation is that we overlooked some OBI cases in HBV-seronegative patients, though, as we mentioned before, this is quite a rare occurrence.

Concerning the detection of HBsAg, recent studies found that between 1% and 48% of samples that tested negative using assays with a limit of detection of 0.05 IU/mL or higher tested positive using more sensitive HBsAg assays with a lower limit of detection of 0.005 IU/mL. Previous observations have confirmed the higher sensitivity of the HBsAg NEXT assay compared to the former version without compromising specificity [7,23], as also shown by the distribution of negative results (Figure S1), and the results compared with HBV-DNA detection were aligned with expectations as samples with a very low HBV-DNA load, i.e., detectable but not quantifiable, tested negative for HBsAg. On the other side, finding four samples that were positive for HBsAg and negative for HBV-DNA suggests that the adoption of HBsAg assays with greater sensitivity may help to better define the pattern of HBV infection and will reduce the frequency of OBI. HBsAg and HBV-DNA have different kinetics both in untreated subjects and in patients treated with antivirals [9,12,21,24], and infectivity from samples positive for HBsAg and negative for HBV-DNA has been documented [25].

## 5. Conclusions

HBV coinfection occurs with a high frequency both in patients with HIV and HCV. While OBI and overt HBV infection were infrequent in HIV-positive subjects, in our observation, patients with an ongoing HCV infection showed a high rate (10.2%) of active HBV infection, which may bear a risk for reactivation, especially when those patients are treated with antivirals. A change in the monitoring profile in patients at high risk for HBV infection and reactivation by testing for both HBV-DNA and HBsAg by highly sensitive assays [26] shall therefore be envisioned.

## Figures and Tables

**Table 1:** Demographic data and testing results on patients belonging to groups A (HIV-infected) and B (HCV-infected). The median HBV-DNA levels were calculated, assigning a value of 20 IU/mL to samples lower than the limit of detection of the assay employed (30 IU/mL). NA, not available; NC, not calculated; *p* values relative to differences between group A and group B were calculated by the chi-square test or Kruskal–Wallis test.

Parameter	Group A: Subjects with HIV	Group B: Subjects with HCV	*p*-Value
Patients, *N*	499	137	*NC*
Male, *n* (%)	418 (83.8)	87 (63.6)	*<0.001*
Age in years, Median (range)	53 (18–83)	54 (19–96)	*0.012*
Log_10_ IU/mL HCV-RNA, median (range)	NA	5.75 (1.00–7.35)	*NC*
Positive for HBV biomarkers, *n* (%)	495 (99.1)	98 (71.5)	*<0.001*
Anti-HBc positive only, *n* (%)	105 (21)	48 (35)	*<0.005*
Anti-HBs positive only, *n* (%)	11 (2.2)	9 (6.6)	*<0.001*
Anti-HBc and anti-HBs positive, *n* (%)	379 (76)	41 (29.9)	*<0.001*
HBV-DNA positive, *n* (%)	3 (0.6)	10 (7.3)	*<0.001*
Log_10_ IU/mL HBV-DNA, median (range)	1.30 (1.30–1.82)	1.30 (1.30–2.12)	*0.70*
Subjects coinfected by HIV, *n* (%)	499 (100)	54 (61.4)	*<0.001*
Subjects with suppressed HIV replication, *n* (%)	414 (80)	39 (72.2)	*0.02*

**Table 2:** Demographic data and testing results on patients belonging to groups A (HIV-infected) and B (HCV-infected). The median HBV-DNA levels were calculated, assigning a value of 20 IU/mL to samples lower than the limit of detection of the assay employed (30 IU/mL). NA, not available; NC, not calculated; *p* values relative to differences between females and males were calculated by the chi-square test or Kruskal-Wallis test.

	Group A: Subjects with HIV		
Parameter	Females	Males	*p*-Value
Patients, *n* (%)	81 (16.2)	418 (83.8)	*0.002*
Age in years, median (range)	53 (25–75)	53 (18–83)	*0.337*
Positive for HBV biomarkers, *n* (%)	80 (98.8)	415 (99.3)	*0.509*
Anti-HBc positive only, *n* (%)	25 (30.8)	80 (19.1)	*0.04*
Anti-HBs positive only, *n* (%)	1 (1.2)	10 (2.4)	*0.08*
Anti-HBc and anti-HBs positive, *n* (%)	54 (66.7)	325 (77.7)	*0.06*
HBV-DNA positive, *n* (%)	0 (0)	3 (0.7)	*1.00*
Log_10_ IU/mL HBV-DNA, median (range)	NA	1.30 (1.30–1.82)	*NC*
Subjects with suppressed HIV replication, *n* (%)	60 (74.1)	354 (84.7)	*0.05*
	**Group B: subjects with HCV**		
**Parameter**	**Females**	**Males**	***p*-Value**
Patients, *n* (%)	50 (36.4)	87 (63.6)	*0.05*
Age in years—median (range)	54 (19–92)	55 (19–96)	*0.43*
Log_10_ IU/mL HCV-RNA, median (range)	5.83 (1.08–7.15)	5.71 (1.00–7.35)	*0.62*
Positive for HBV biomarkers, *n* (%)	28 (56.0)	70 (80.4)	*0.002*
Anti-HBc positive only, *n* (%)	14 (28)	34 (39.1)	*0.19*
Anti-HBs positive only, *n* (%)	5 (10)	4 (4.6)	*0.22*
Anti-HBc and anti-HBs positive, *n* (%)	9 (18)	32 (36.8)	*0.02*
HBV-DNA positive, *n* (%)	4 (8)	6 (6.9)	*1.00*
Log_10_ IU/mL HBV-DNA, median (range)	1.30 (1.30–1.30)	1.68 (1.30–2.12)	*0.06*
Subjects coinfected by HIV, *n* (%)	13 (52)	41 (65.1)	*0.26*
Subjects with suppressed HIV replication, *n* (%)	9 (69.2)	30 (73.2)	*0.78*

**Table 3:** Mean features for samples positive for HBV-DNA and/or HBsAg NEXT on the total study population. The capital letter in subject IDs indicates the group to which each sample belongs: A, samples from patients with HIV (light grey background); B, samples from patients with HCV (white background); C, samples with low HBV-DNA levels (dark grey background). Samples C6 and C8 were positive by the HBV-DNA and HBsAg NEXT assays. Pos, positive; Neg, negative; BL, borderline positive; NA, not available.

Subject ID	Age, Years	Gender	Anti-HBc	Anti HBs	HBV-DNA, IU/mL	HBsAg NEXT	HBsAg NEXT, S/CO	HIV Seropositivity	HIV Status According to the HIV-RNA Levels
A91	56	M	Pos	Neg	<30	Neg	0.37	Pos	Not suppressed
A358	55	M	Pos	Pos	67	Neg	0.42	Pos	Suppressed
A370	42	M	Pos	Neg	<30	Neg	0.44	Pos	Suppressed
B6	43	M	Pos	Neg	Neg	Pos	1.03	Pos	Not suppressed
B32	59	F	Pos	Neg	<30	Neg	0.39	Pos	Not suppressed
B34	82	F	Neg	Neg	<30	Neg	0.86	NA	NA
B36	40	M	Pos	Pos	133	Neg	0.55	NA	NA
B37	70	F	Neg	Neg	<30	Neg	0.43	NA	NA
B38	38	M	Pos	Neg	48	Neg	0.37	Pos	Suppressed
B40	52	M	Pos	Pos	72	Neg	0.70	NA	NA
B43	57	F	Pos	Neg	Neg	Pos	1.98	Pos	Suppressed
B53	28	M	Pos	Pos	Neg	Pos	1.05	Pos	Suppressed
B57	65	M	Pos	Neg	<30	Neg	0.33	Pos	Suppressed
B66	61	M	Pos	Pos	Neg	Pos	1.24	Neg	NA
B81	56	M	Pos	Neg	<30	Neg	0.37	NA	NA
B140	66	F	Pos	Neg	<30	Neg	0.5	NA	NA
B151	51	M	Pos	Neg	116	Neg	0.49	Pos	Not suppressed
C1	54	M	Pos	Neg	64	Neg	0.61	Pos	Not suppressed
C2	45	M	Pos	Neg	<30	Neg	0.46	Neg	NA
C3	52	F	Pos	Pos	11	Neg	0.31	Pos	Not suppressed
C4	49	M	Pos	Neg	<30	Neg	0.42	Pos	Not suppressed
C5	62	M	Pos	Pos	<30	Neg	0.42	Neg	NA
C6	32	M	Pos	Pos	39	Pos	4.28	Pos	Suppressed
C7	46	F	Pos	Neg	40	Neg	0.40	Neg	NA
C8	57	F	BL	Neg	1186	Pos	4.55	Pos	Not suppressed

## Data Availability

The data presented in this study are available on request from the corresponding author.

## Supplementary Materials

The following supporting information can be downloaded at: https://www.mdpi.com/article/10.3390/v16030412/s1, Figure S1: Frequency of sample-to-cutoff distribution of HBsAg NEXT negative results in the whole study cohort; Table S1: Demographic data and testing results on patients belonging to group C (subjects with low HBV-DNA).
