# Supplementary figures and images for: Occult HBV Infection in Patients Infected by HIV or HCV: Comparison between HBV-DNA and Two Assays for HBsAg
Source: Viruses. 2024 Mar 7;16(3):412. doi: 10.3390/v16030412 (PMC10974054; doi:10.3390/v16030412)

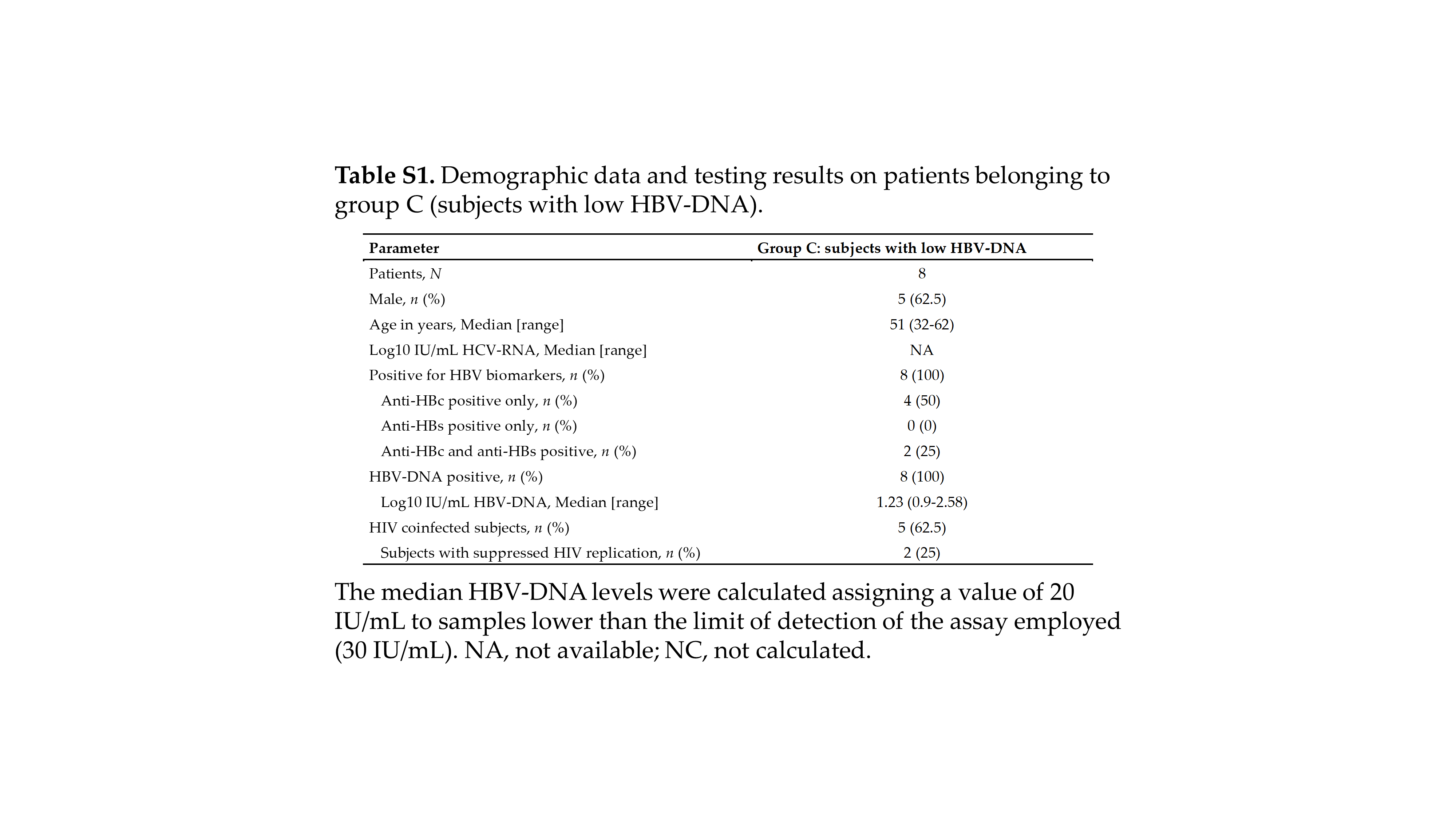

Supplement: Supplementary file 1 [file viruses-16-00412-s001.zip › viruses-2893189 v1 Table S1 and Figure S1 06.03.24/Diapositiva1.TIF]

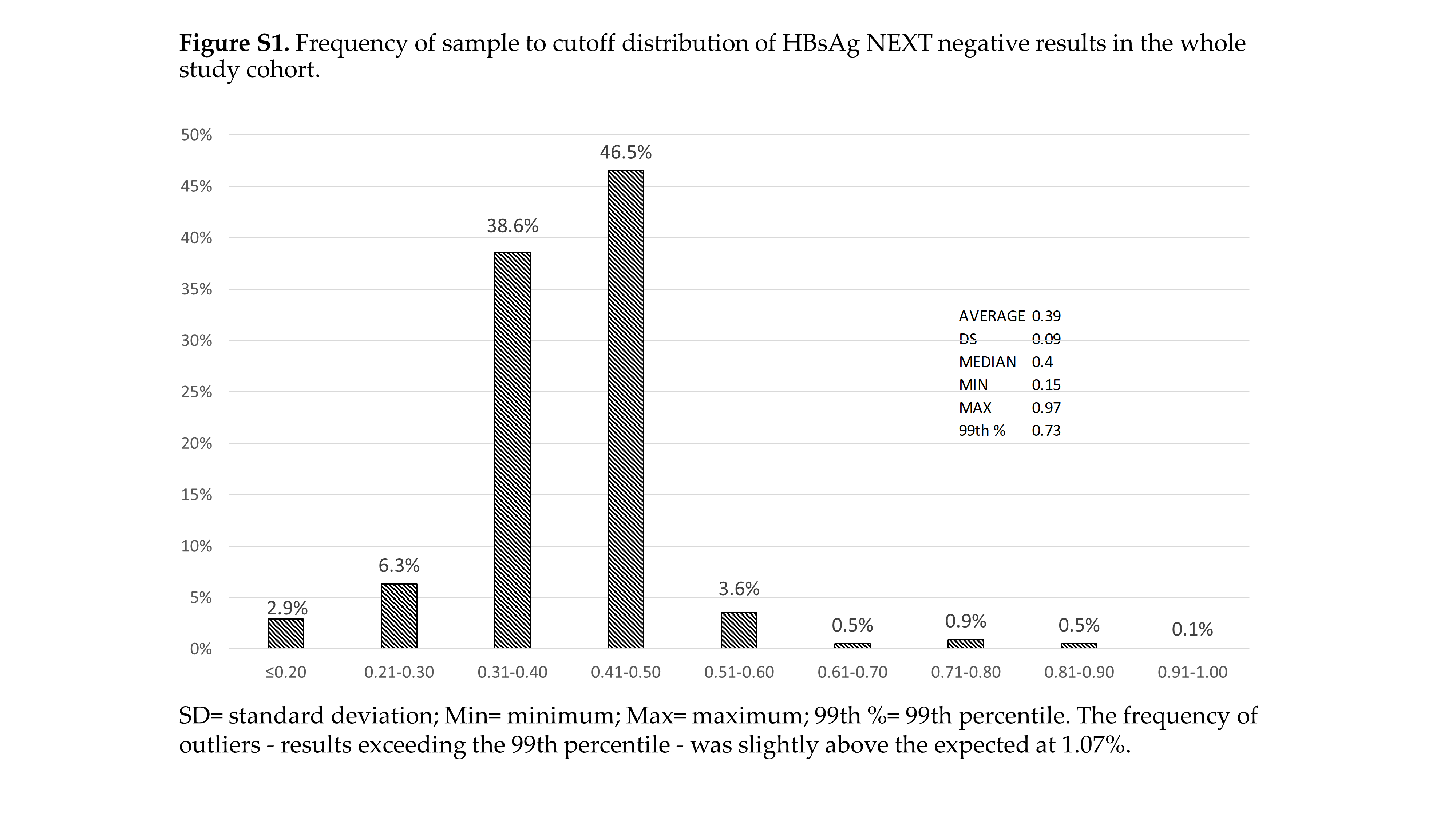

Supplement: Supplementary file 1 [file viruses-16-00412-s001.zip › viruses-2893189 v1 Table S1 and Figure S1 06.03.24/Diapositiva2.TIF]
